# A 71‐year‐old female with an intrasellar mass

**DOI:** 10.1111/bpa.12960

**Published:** 2021-06-21

**Authors:** Yanli Du, Taili Li, Hui Qi, Zongli Han

**Affiliations:** ^1^ School of Medical Technology and Nursing Shenzhen Polytechnic Shenzhen P.R. China; ^2^ Department of Pathology Peking University Shenzhen Hospital Shenzhen P.R. China; ^3^ Department of Neurosurgery Peking University Shenzhen Hospital Shenzhen P.R. China

**Keywords:** intrasellar cavernous hemangioma

## Abstract

Magnetic resonance imaging revealed an intrasellar and suprasellar tumor with significant homogeneous contrast enchancement (Figure A). Under microscopy showed the dilated cavernous spaces of irregular size and shape, which are embedded in the loose collagenous matrix and adipose tissue, lined by normal flattened endothelial cells. These spaces are mostly empty and some cavities are filled with proteinaceous material (Figure B). This mass is immunohistoreactive for CD34 (Figure C) and D2‐40 (Figure D).

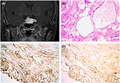

**BOX 1 Slide scan**

Access the whole slide scan at http://image.upmc.edu:8080/NeuroPathology/BPA/BPA-20-12-301.svs/view.apml (the extraction code is 1234).

## 1 CLINICAL HISTORY

**FIGURE 1:**
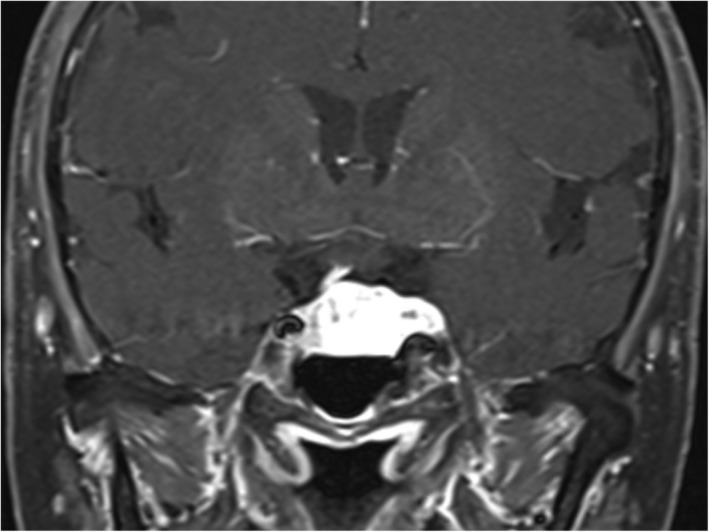
Coronal T1‐weighted contrast MRI showed a homogeneous enhancement of sellar and suprasellar mass

A 71‐year‐old female was admitted with a left eye decreased visual acuity for more than 2 years. Preoperative basal values of thyroid‐stimulating hormone, growth hormone, luteinizing hormone, follicle‐stimulating hormone, cortisol, and adrenocorticotropic hormone were unremarkable except for a slightly increased prolactin. Computer tomography showed an enlarged sella turcica and isoattenuated sellar lesion. Magnetic resonance imaging (MRI) revealed an enlarged sellar turcica and sellar mass with suprasellar and parasellar extension. Coronal T1‐weighted MRI showed a predominantly isointense to cerebral cortex circumscribed sellar/suprasellar mass. Coronal T2‐weighted image showed a high signal of the tumor. A significant homogeneous increase in the signal intensity of the tumor was showed by contrast enhancement (Figure 1). The preliminary diagnosis of non‐function pituitary adenoma was made. A transsphenoidal approach was selected for the removal of the sellar mass. We noticed a red‐tan and vascular fibrous tumor with a slightly tougher consistency with cystic degeneration than that of usual pituitary adenomas. The tumor was removed incompletely because of its tight consistency.

## 2 FINDINGS

**FIGURE 2:**
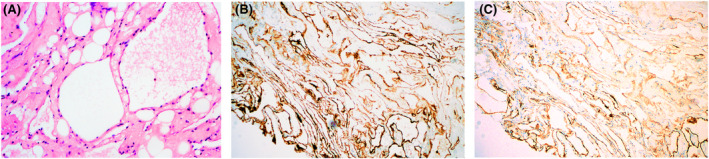
(A) Histopathologic examination of the tumor (hematoxylin and eosin stain; magnification ×200) showed the dilated cavernous spaces of irregular size and shape, which are embedded in the loose collagenous matrix and adipose tissue, lined by normal flattened endothelial cells showing no nuclear atypia. These spaces are mostly empty and some cavities are filled with proteinaceous material. (B) Immunohistoreactive for CD34 (magnification ×100). (C) Immunohistoreactive for D2‐40 (magnification ×100)

Photomicrograph of the operative tumor specimen showed the dilated cavernous spaces of irregular size and shape, which are embedded in the loose collagenous matrix and adipose tissue without lymphoid aggregates, lined by normal flattened endothelial cells showing no nuclear atypia. These spaces are mostly empty and some cavities are filled with proteinaceous material (Figure 2A). This mass is immunohistoreactive for CD34 (Figure 2B) and D2‐40 (Figure 2C).

## 3 DIAGNOSIS

Intrasellar cavernous hemangioma.

## 4 DISCUSSION

Extra‐axial cavernous hemangiomas are most frequently seen in the fourth and fifth decades of life with a substantial female prevalence of about 7:1 and are more prevalent in the Asian population especially Japanese which have different clinical and imaging features compared with the more common intra‐axial cavernous hemangiomas. The term “intrasellar cavernous hemangiomas” was used to refer to cavernous hemangiomas mainly or soundly involving the sella. The authors believe it is a type of cavernous sinus hemangiomas growing into the sella which may originate from the components of the cavernous sinus itself or the surrounding tissues.

Because of its rarity, little published information regarding the pathogenesis of this entity is available. Some studies (1, 2) suggest that hemangiomas may be due to dysregulated stem cells that remain in an immature arrested stage of development. They found that endothelial cells from proliferating hemangiomas are a result of clonal expansion and differ from normal endothelial cells in their rates of proliferation and migration. Furthermore, the migration of hemangioma endothelial cells was stimulated by the angiogenesis inhibitor endostatin, in contrast to inhibition which is seen with normal endothelial cells. This phenomenon suggests the possibility of an altered cellular phenotype.

Pathologically, intra‐axial hemangioma often demonstrates hemosiderin‐laden macrophages, which usually are small and exhibit multiple vascular channels in various states of thrombosis, which likely reflects the low flow nature of these lesions. Extra‐axial counterparts, however, can be large and can contain vascular channels that lack histological evidence of thrombosis and calcification, which are consistent with their high‐flow state. Intra‐axial pathology is thought to represent true vascular malformations, which grow in response to intralesional microhemorrhage, whereas extra‐axial disease is believed to constitute benign vascular tumors, with intrinsic growth of endothelial cells. Based on both pathological and clinical differences, extra‐axial cavernous hemangiomas have been categorized into two subtypes. Subtype A is reported to be associated with severe intraoperative bleeding. Pathologically, these lesions exhibit thin‐walled, sinusoidal, adjacent vessels with scant intervening connective tissue. In contrast, subtype B lesions have more intervening connective tissue than subtype A. The vessels of subtype B are less sinusoidal than those of ​subtype A, and their size and shape are more variable and irregular.

Interestingly, our case showed both vascular and lymphatic endothelial phenotypes based on their immunoreactivity with D2‐40 and CD34. Initially, we made a pathological diagnosis of lymphangioma based on a positive response to D2‐40, but this vascular lesion does not show the presence of lymphoid aggregates in the stroma typical of lymphangioma. One explanation might be that D2‐40, which was considered specific for lymphatic vessels can be expressed in other vascular lesions and is not sufficient to accurately differentiate a lymphangioma. Another potential explanation might be that the tumor cells derive from stem cells possessing the characteristics of both lymphatic and blood vessel endothelial lineages.

Despite their benign nature, these lesions are sometimes difficult to treat because of their tendency to be poorly demarcated, to be infiltrative, bleeding prone, and to involve vital structures.

## CONFLICT OF INTEREST

The authors declare that they have no conflict of interest.

## AUTHOR CONTRIBUTIONS

Yanli Du: drafting the manuscript, acquisition of data. Taili Li: Interpretation of pathological results. Hui Qi: drafting and revising the manuscript. Zongli Han: study design, supervision of study.

## ETHICAL STANDARDS AND PATIENT CONSENT

We declare that all human and animal studies have been approved by the Peking University Shenzhen Hospital ethics committee and have therefore been performed in accordance with the ethical standards laid down in the 1964 Declaration of Helsinki and its later amendments.

## INFORMED CONSENT

Informed consent was obtained from all individual participants included in the study.

## Data Availability

The data that support the findings of this study are available from the corresponding author upon reasonable request.
